# Toll-like receptor 4 in glial inflammatory responses to air pollution in vitro and in vivo

**DOI:** 10.1186/s12974-017-0858-x

**Published:** 2017-04-14

**Authors:** Nicholas C. Woodward, Morgan C. Levine, Amin Haghani, Farimah Shirmohammadi, Arian Saffari, Constantinos Sioutas, Todd E. Morgan, Caleb E. Finch

**Affiliations:** 1 Leonard Davis School of Gerontology, University of Southern California, Los Angeles, CA USA; 2 Department of Human Genetics, David Geffen School of Medicine, University of California Los Angeles, Los Angeles, CA USA; 3 Viterbi School of Engineering, University of Southern California, Los Angeles, CA USA; 4 Dornsife College, University of Southern California, Los Angeles, CA USA

**Keywords:** Air pollution, Nanoparticulate matter, Microglia, Astrocytes, TLR4, NF-kB, TNFα, Cell culture, Hippocampus

## Abstract

**Background:**

Exposure to traffic-related air pollution (TRAP) is associated with accelerated cognitive aging and higher dementia risk in human populations. Rodent brains respond to TRAP with activation of astrocytes and microglia, increased inflammatory cytokines, and neurite atrophy. A role for Toll-like receptor 4 (TLR4) was suggested in mouse TLR4-knockouts, which had attenuated lung macrophage responses to air pollution.

**Methods:**

To further analyze these mechanisms, we examined mixed glial cultures (astrocytes and microglia) for RNA responses to nanoscale particulate matter (nPM; diameter <0.2 μm), a well-characterized nanoscale particulate matter subfraction of TRAP collected from a local freeway (Morgan et al. Environ Health Perspect 2011; 119,1003–1009, 2011). The nPM was compared with responses to the endotoxin lipopolysaccharide (LPS), a classic TLR4 ligand, using Affymetrix whole genome microarray in rats. Expression patterns were analyzed by significance analysis of microarrays (SAM) for fold change and by weighted gene co-expression network analysis (WGCNA) to identify modules of shared responses between nPM and LPS. Finally, we examined TLR4 activation in hippocampal tissue from mice chronically exposed to nPM.

**Results:**

SAM and WGCNA analyses showed strong activation of TLR4 and NF-κB by both nPM and LPS. TLR4 siRNA attenuated TNFα and other inflammatory responses to nPM in vitro, via the MyD88-dependent pathway. In vivo, mice chronically exposed to nPM showed increased TLR4, MyD88, TNFα, and TNFR2 RNA, and decreased NF-κB and TRAF6 RNA TLR4 and NF-κB responses in the hippocampus.

**Conclusions:**

These results show TLR4 activation is integral in brain inflammatory responses to air pollution, and warrant further study of TLR4 in accelerated cognitive aging by air pollution.

**Electronic supplementary material:**

The online version of this article (doi:10.1186/s12974-017-0858-x) contains supplementary material, which is available to authorized users.

## Background

Traffic-related air pollution (TRAP) is associated in human populations with accelerated cognitive aging [1–4] and increased risk of dementia [5–8]. Exposure to high levels of fine particulate matter (PM_2.5_) in older adults is associated with greater cognitive decline, equivalent to 2 years of normal cognitive aging [9, 10], and developmental exposure to TRAP is associated with delayed and impaired cognitive development [11, 12].

TRAP-associated changes of normal aging include decreased white and grey matter [13, 14], while post mortem samples from a highly polluted Mexican City showed white matter hyperintensities and neuroinflammation [15, 16]. Rodent models given controlled exposure to TRAP particulate material (PM), showed corresponding loss of dendritic spines [17] and microglial activation [18, 19]. Brain inflammatory responses include increased IL-1α and TNFα [17, 18, 20], together with NF-κB [21] and detoxifying enzymes associated with Nrf2 [22]. In our model, mice were chronically exposed to nPM, a nano-scaled subfraction of TRAP [18, 23].

Airborne PM is defined by three size classes: coarse, PM_10_; <10 μm diameter; fine, PM_2.5_; <2.5 μm; and ultrafine, PM_0.2_; <0.2 μm. The PM_0.2_, though not EPA regulated, have more toxicity and greater redox activity than larger size fractions [24]. In rodents, in vivo exposure to PM_0.2_ shows their penetration into the brain via the nose [25, 26]. The present studies use a nano-sized water-soluble subfraction of PM_0.2_ (nPM) with strong redox and inflammatory activity in vitro and in vivo that induced microglial activation TNFα and other cytokines associated with oxidative stress [24, 27].

To further study glial roles, we used an in vitro model of mixed glia (astrocytes and microglia), in which nPM induced TNFα induction with well-defined dose response [27]. In media from nPM-treated glia, primary neurons had shorter neurites and fewer growth cones; these nPM effects were mediated by the TNFα receptor TNFR1 [27]. We hypothesized further that these mechanisms involve a priori genes of interest NF-κB and TLR4 (Toll-like receptor 4), through MyD88-dependent pathways, shown for lung macrophage responses to TRAP [28, 29]. TLR4 has over 30 known ligands [30]. Its role in inflammatory pathways may include neuroprotective functions, e.g., by increasing amyloid β-peptide uptake by microglia [31]. While a TLR4 response to air pollution has been hypothesized, its exact involvement remains unclear, as TLR4 mutant individuals still showed immune cell response to PM [16].

These questions were agnostically approached by Affymetrix microarray in mixed glia cultures for responses to nPM and to lipopolysaccharide (LPS). The endotoxin LPS is relevant to TRAP induced inflammatory responses. While nPM does not contain endotoxin [32], the larger sized PM have endotoxin activity [33], whereas the TLR4 pathway is necessary for LPS responses. Our findings were compared with LPS responses of BV-2 microglial cells [34, 35] and primary cultured microglia [36].

Two bioinformatics approaches were used: fold changes in RNA expression were identified by significance analysis of microarrays (SAM) and shared modules of RNA responses between LPS and nPM were identified by weighted gene co-expression network analysis (WGCNA), which has advantages over differential expression analysis. WCGNA enables the reduction of high-dimensional data into fewer variables for analysis of shared responses between treatments. While both SAM and WGCNA identify larger responses, WGCNA also identifies subtler interactions within a network. WGCNA provides further insight into the relationships between genes, extending beyond known pathways [37].

Following SAM and WGCNA, we analyzed transcription factor target (TFT) enrichment and upstream regulators. Finally, bioinformatics findings were verified in vitro by siRNA experiments with mixed glia and in vivo with hippocampal RNA from mice exposed to nPM.

## Methods

### Animals and ethics statement

Pregnant Sprague Dawley rats, from Envigo (Livermore, CA, USA) and C57BL/6J female mice, from the NIA Aged Rodent Colony (Charles River Labs) were maintained under standard conditions according to NIH guidelines. Protocols were approved by the University of Southern California Institutional Animal Care and Use Committee.

### Collection of nanoscale particulate material (nPM)

The nPM utilized in these studies are a nanoscale subfraction of TRAP (<200 nm diameter) collected from urban air in Los Angeles near the CA-110 Freeway using a high-volume impactor sampler [38]. These aerosols represent a mix of fresh ambient PM mostly from vehicular traffic [39, 40]. nPM was collected continuously for 5 weeks on Teflon filters, followed by resuspension in deionized water by vortexing and sonication [18]. The nPM comprised approximately 20% by mass of ambient PM_2.5_ in that location [41]. Water-soluble metals and organic compounds were efficiently transferred from the filter into aqueous suspension for exposures [18]. Relative to the total filter-trapped ultrafines (PM_0.2_), the nPM subfraction eluted into aqueous phase was depleted in black carbon and water-insoluble organic compounds [18]. Stock nPM solution had trace endotoxin levels (2.5 EU/mL by *Limulus* amoebocyte assay) compared to that eluted from filter collected ambient air (2.0 EU/mL). Treatment levels were 0.05–0.08 EU/mL, equivalent to sterile water (FDA 2015). These low EU levels are consistent with the negligible endotoxin activity in ultrafine PM (below *Limulus* assay threshold) [32]. Frozen stocks at 20 °C retain chemical stability for >30 days, including long-lived free radicals [18, 24]. The nPM was re-aerosolized to an airborne concentration of 300 μg/ml. Mice (3 month old) were exposed to nPM or filtered air for 150 h during 10 weeks (5 h/day, 3 days/week).

### Tissue collection

One month after exposure, mice were anesthetized by isoflurane and saline perfused. One hemisphere was cryosectioned for histochemistry and the other, microdissected by brain region.

### Cell culture

Primary mixed glia (microglia and astrocytes) were cultured from neonatal postnatal day 3 rat cerebral cortex (mixed sex, Sprague Dawley; Envigo, Livermore, CA). Mixed glial cultures were used because some astrocyte responses to LPS are microglial dependent [42, 43]. Mixed glial cultures were 3:1 astrocytes to microglia [27]. Cerebral cortex was mechanically dissociated, strained by a 70-μm Millipore filter, and plated onto 75-cm^2^ cell culture flasks in Dulbecco’s modified Eagle’s medium/F12 (Cellgro, Mediatech, Herndon, VA) supplemented with 10% fetal bovine serum, 1% penicillin, and 1% L-glutamine. Cultures were incubated at 37 °C with 95:5% mixture of air:CO_2_. Media was refreshed twice in week 1 and once during week 2. Cells were trypsinized, plated on six-well plates, and treated with nPM (10 μg/mL, 24 h) or LPS (100 ng/mL, 48 h for microarray, 24 h for TLR4 knockdown). The 10 μg/mL dose of nPM is based on published work demonstrating conditioned media from mixed glia treated with 10 μg/mL nPM reduced inhibited neurite outgrowth and reduced the number of neurites [18].

### RNA

Total cell RNA was isolated by TRIzol; cDNA was reverse transcribed (Promega) and analyzed on Affymetrix Rat Whole Genome 230.2 array. For individual gene verification, q-PCR was done with Taq Master Mix (Biopioneer), with primers designed with Primer3 and verified by NCBI Primer-BLAST (Basic Local Alignment Search Tool). Data was quantified as ΔΔCT and normalized to GAPDH.

### Microarray analysis

For nPM microarray, RNA-cDNA was prepared from *N* = 4 treated and *N* = 4 controls. For LPS cultures, we compared five treated versus four control cultures. Microarray was verified by q-PCR, with *N* = 14 per group.

#### Normalization

Raw Affymetrix data were normalized by the Robust Multi-array Averaging R algorithm [44–46]. The output is then transformed to log_2_. LPS and nPM microarrays were independently normalized to controls within each individual experiment. These datasets are available as raw data files, given in Additional file 1.

#### Significance analysis of microarrays (SAM)

Normalized microarray data were analyzed for fold change by SAM, to assess differential expression associated with nPM and/or LPS exposure relative to controls. The input into SAM was an individual log_2_ fold change score for each microarray probe, with significance calculated using permutation tests (100 permutations) and presented as *q*-values to account for false discovery rate, with an FDR threshold at 1% (*q* < 0.01).

#### Weighted gene co-expression network analysis (WGCNA)

Gene expression networks were identified using the WGCNA package in R [47]. Adjacency matrices for nPM and LPS were generated by first calculating biweight midcorrelations of gene expression for each gene pair. To construct weighted gene networks, these measures of co-expression (correlation coefficients) are raised to a power of *β*—the soft-thresholding power. This value is chosen by analyzing scale-free topology. More information on selecting a *β* value can be found in work by Zhang and Horvath [48]. For this analysis we used a soft-thresholding power of 6. Adjacency for each gene pair was then defined using topological overlap matrices (TOMs). TOM incorporates higher-order connections by taking into account the number of “neighbors”, or connections, that a pair of genes share [49]. Individual TOMs were estimated for both nPM and LPS. A consensus topological overlap (across nPM and LPS) was calculated using the component-wise (parallel) minimum of the individual TOMs. TOMs were then converted to dissimilarity matrices by subtracting from 1. Modules were then defined by employing hierarchical clustering with TOMs as input with the following WGCNA parameters: unsigned network, minimum module size of 30, and medium sensitivity (deepSplit = 2). Hierarchical clustering assigns each gene to a specific module (denoted by different colors), which represent networks of highly interconnected genes.

Following module assignment, eigengenes were estimated for each module. The eigengene is a single quantitative value for a module, which represents its overall gene score, based on levels of messenger RNA (mRNA) for all genes assigned to that module. The use of eigengenes enables dimension reduction—instead of comparing gene expression between exposure and controls for thousands of genes, comparisons of higher-order measures of gene expression between exposure and controls can be carried out for less than 100 modules. We then screened for hub genes (genes that are highly connected *within* a given module). Although the hierarchical clustering assigns each gene to one module, module membership can be defined by calculating eigengene-based connectivity (kME) for each gene-by-module pair as the correlation between expression levels of a gene and the eigengene (quantitative score) for a module. kME was calculated in each set (nPM and LPS), then consensus kME scores were generated as meta-analytic scores by Stouffer’s method. Based on kME, hubs for each module were defined as the genes with the highest connectivity (kME ≥0.80).

#### Pathway analysis

Pathway enrichment analysis by Kyoto Encyclopedia of Genes and Genomes (KEGG) (www.genome.jp/kegg/) and Gene Ontology (GO; Gene Ontology Consortium, geneontology.org) used two gene sets as inputs—those with differential expression for both the nPM and LPS treatment (SAM results), and those identified as hub genes in modules enriched by both nPM and LPS (WGCNA results). Whereas GO was utilized in SAM to determine only the most enriched responses, GO is utilized here to describe the composition of the module and therefore uses relaxed stringency. Increased and decreased transcripts were considered independently [50]. Pathway figures were generated using Cytoscape with the ClueGo plugin [51], with enriched processes depicted as nodes, and networks, which are groups of connected nodes.

Genes significant by SAM from each dataset, and shared hub genes from modules enriched by both treatments were analyzed for enriched transcription factor targets (TFTs) using WEB-based Gene Set Analysis Toolkit (WEB-GESTALT); cutoff at *p <* 0.01. Because TFT analysis makes corrections for false positives, TFT analysis has more power in connected networks (WGCNA modules) versus broad gene lists (SAM results) and can therefore detect greater TFT enrichment in a subset versus complete gene lists.

### TLR4 siRNA cultures

Glia were transfected with lipofectamine (10 μL/well media) and TLR4 siRNA (Thermo Fisher Scientific, siRNA ID 198667), or negative control siRNA (Thermo Fisher Scientific, AM4611), combined with Opti-MEM media and added to culture media. For control cultures, only Opti-MEM media was added to culture media. After 48 h, cells were treated with either nPM (10 μg/mL, 24 h) or LPS (100 ng/mL, 24 h), and harvested for RNA (TRIzol) and protein (RIPA buffer).

Scrambled RNA induced JNK1/2 (no change by nPM, 1.6× by scrambled RNA) and STAT1 (4.7× by nPM, 7.4× by scrambled RNA), with no effect on other genes queried. nPM effects were unchanged by scrambled RNA.

#### Inflammatory proteins

Protein concentration was analyzed by the V-PLEX Proinflammatory Panel 2 immunoassay (K15059D-1, Mesoscale Diagnostics, Rockville, MD).

### Statistical analysis

Ordinary one-way analysis of variance test was used, with Tukey’s posttest to correct for multiple comparisons. All analysis used Graphpad Prism 6.

### Heatmap

Hierarchical clustering and heatmap generation were performed in MatLab (Version R2014b) to identify the relation of gene expression changes among different groups of this study. Genes were clustered by Pearson correlation coefficients.

## Results

### Strategy

**Fig. 1:**
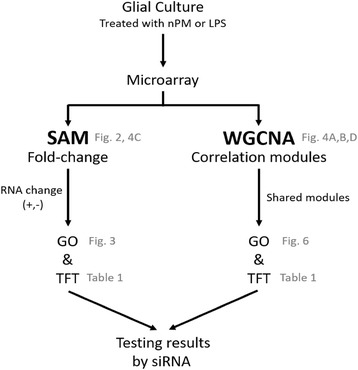
Flowchart of analysis. RNA responses of mixed glial cultures to nPM or LPS were analyzed by whole genome microarray for fold change by significance analysis of microarrays (SAM), and for shared RNA responses by weighted gene co-expression network analysis (WGCNA), given as correlation modules. Correlation modules are the clusters of correlated genes. The results from SAM and WGCNA were then analyzed for biological processes using the Gene Ontology (GO) database, and transcription factor targets (TFTs). GO output is given as nodes, which are enriched biological processes, and as networks, which are groups of connected nodes. Responding RNAs that met the criteria for fold change were analyzed for GO and TFT enrichment. For WGCNA, only modules of shared responses between nPM and LPS were examined

Mixed glial acute responses to nPM and LPS were analyzed by whole genome microarray and processed in tandem by significance analysis of microarrays (SAM), and by weighted gene co-expression network analysis (WGCNA) (Fig. 1). SAM identified responses in individual RNAs, by fold change, to nPM or LPS. WGCNA determined shared RNA responses between nPM and LPS treatments, defined as modules for groups of RNAs with correlated expression. The results from SAM and WGCNA were then analyzed for enriched biological processes, by Gene Ontology (GO), and for transcription factor targets (TFTs).

Guided by results from microarray analysis, we investigated TLR4 involvement in nPM response by TLR4 knockdown in vitro, and investigated TLR4 activation in the hippocampus following chronic in vivo exposure.

#### Significance analysis of microarrays (SAM)

**Fig. 2:**
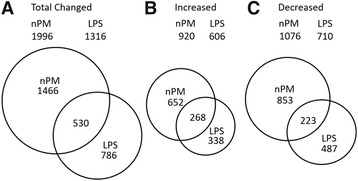
Venn diagram of RNA by significance analysis of microarrays (SAM). Areas of each *circle* are in proportion to gene numbers. **a** Total RNAs altered by either treatment. **b** Increased RNAs by either treatment. **c** Decreased RNAs by either treatment

The nPM treatment altered 1996 RNAs, with 920 increased and 1076 decreased (Venn diagrams, Fig. 2a–c; *q* < 0.1). This analysis only considered RNA changes with *q*-values of 0.01 and with minimum changes of 1.5× for increases and of 0.7× for decreases. LPS induced 1316 RNA responses, with 606 increased and 710 decreased. nPM altered 50% more RNAs than LPS, for both the increased and decreased.

Among the RNA responders to LPS and nPM, 530 were shared: 268 increased in both; 223 decreased in both (Fig. 2a–c; *q* < 0.1). A subset of 39 RNAs had divergent directions of response to nPM and LPS, with 80% increased by nPM. Divergent responses were analyzed by GO (“SAM biological processes analysis by Gene Ontology (GO)”).

#### Verification by q-PCR

Eight TNFα-associated RNA level changes detected by microarray were confirmed by q-PCR: five increased (Jak2, STAT1, TNFα, TNFRSF9, and TRAFD1) and three non-changers (Fos, TRAF3ip, and TRAF6) (Additional file 2: Figure S1).

#### SAM biological processes analysis by Gene Ontology (GO)

The top enriched biological processes (20% minimum enrichment) by nPM or LPS were determined from GO.

**Fig. 3:**
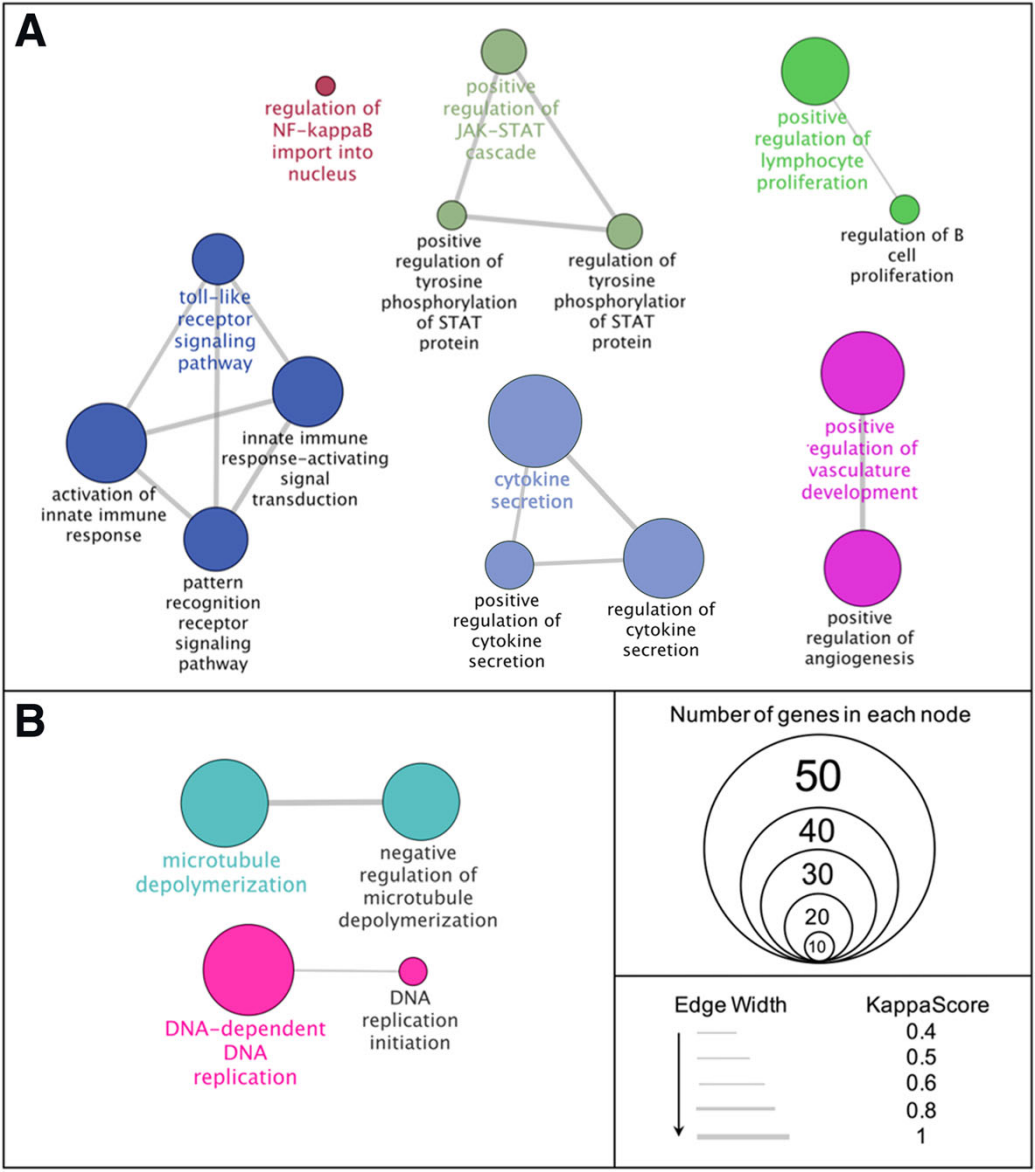
GO processes for nPM responses, analyzed by significance analysis of microarrays (SAM). **a** nPM-increased RNAs: (920), clustered into networks. Note the single node of NF-κB regulation, and TLR signaling pathway network. The cellular migratory network was condensed from 13 nodes to 5. Migratory pathways for T cells and other leukocytes identified by GO analysis are not depicted. **b** nPM-decreased RNAs (1076): Although 17% more RNAs were decreased than the increases, there were fewer enriched pathways. *p <* 0.00001. Colors denote different networks. Nodes with two colors belong to both networks. Each network has one highlighted node (colored text, chosen by experimenter) that best represents network function. The circle size represents the number of genes enriched in the node. The width of connecting lines represents the strength of connectivity between nodes, as measured by kappa score

*nPM responses*: Increased RNAs were associated with 28 GO processes (depicted as nodes, Fig. 3a), including genes of a priori interest NF-κB and TLR4 (“Background”). “Regulation of NF-κB import into nucleus” was represented as one node. Multiple node networks included “Toll-like receptor signaling pathway” (four nodes), “cytokine secretion” (three nodes), and “positive regulation of JAK-STAT cascade” (three nodes). Not depicted in Fig. 3a are “cell migration” nodes, which were enriched for migratory processes of neutrophils, granulocytes, and T cells. These were excluded because of overlap in “migratory” genes between cell types. Decreased RNAs from nPM treatment had two GO networks, each with two nodes: “DNA-dependent DNA replication” and “microtubule depolymerization” (Fig. 3b).

KEGG pathway analysis corroborated the main GO findings: increased RNAs for enrichment in NF-κB, TLR signaling, and cytokine response; decreased RNAs, for enrichment in DNA replication (not shown).

*LPS responses*: Increased RNAs yielded 18 enriched nodes, including “response to tumor necrosis factor”, “I-kappaB kinase/NF-kappaB signaling”, “response to oxidative stress”, “reactive oxygen species metabolic process”, “chronic inflammatory response”, “response to interferon gamma”, “nitric oxide biosynthetic process”, and “response to cytokine” (Additional file 2: Figure S1). Unlike nPM GO, LPS had enriched processes for “antigen processing and presentation of peptide antigen” and “reactive nitrogen species metabolic processes”, among others. Decreased RNAs were enriched for “regulation of protein serine/threonine kinase activity” and “response to wounding” (not shown).

Data from prior microarray analysis of LPS-treated BV-2 microglial cells [34, 35] and primary cultured microglia [36] were also analyzed for GO processes. These datasets showed LPS responses of NF-κB, IFN-y, oxidative stress, and cytokine pathways, corresponding to our results.

*Divergence between nPM and LPS*: For the 39 RNAs with divergent responses by SAM, identified above (“Significance analysis of microarrays (SAM)” section), one third (15/39) were in the “response to chemical stimulus”.

#### Weighted gene co-expression network analysis (WGCNA)

**Fig. 4:**
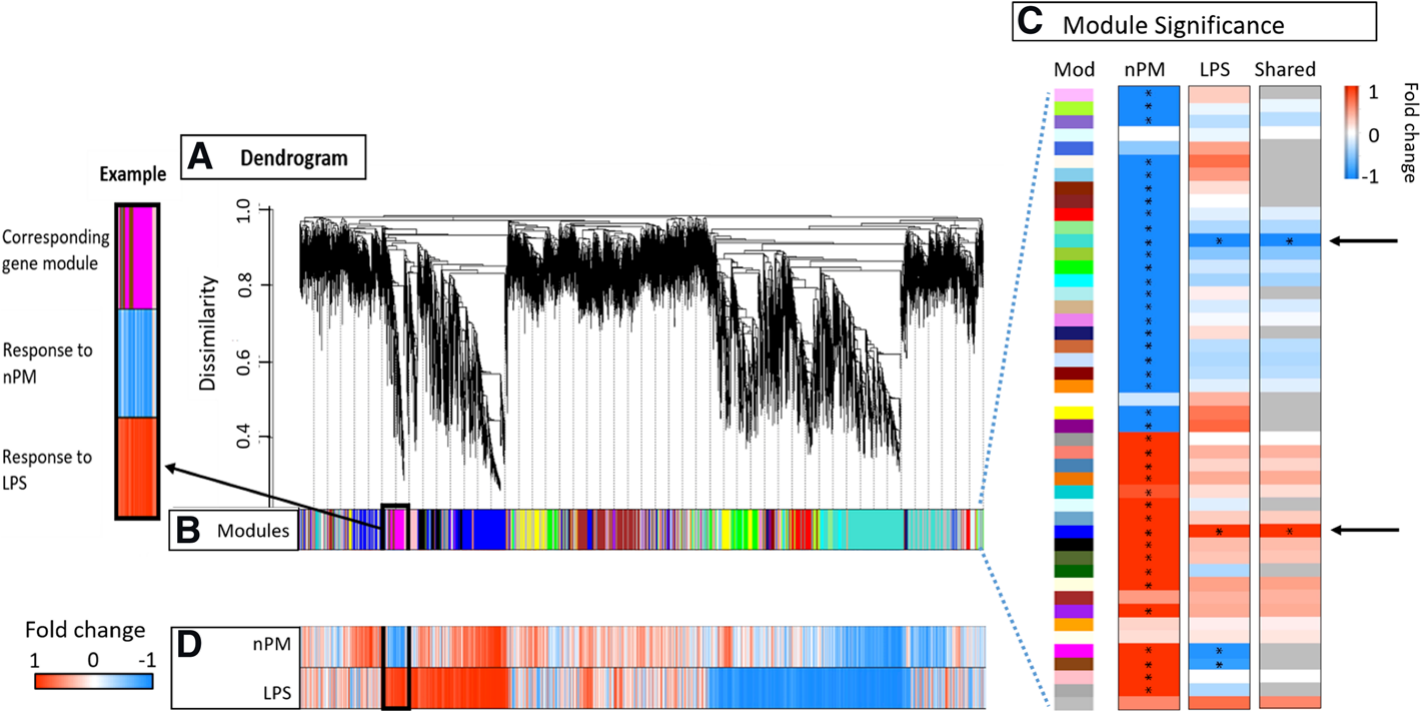
Weighted gene network co-expression analysis (WGCNA) of nPM and LPS responses, grouped as modules. **a** Dendrogram using hierarchical clustering to group genes by lowest values generated by a dissimilarity matrix. **b** Modules, color labeled for each gene (arbitrary colors), with modules placed by highest similarity between gene clusters. 45 modules were generated by WGCNA. **c** Statistical significance of each weighted gene co-expression network analysis (WGCNA) module, for nPM, LPS, and shared responses. Module colors from Fig. 4b are in the *leftmost column* (Mod). Modules from Fig. 3 with significant change (*p <* 0.01) are denoted by *. Of these 45 modules, 38 were enriched in the nPM subset, 4 in LPS subset, and 2 in the shared subset. Shared modules, which will be further analyzed for pathway enrichment, are marked by *arrows*. Scale bar is correlation coefficients between module eigengene and treatment condition. **d** Normalized fold change of RNA for each gene from nPM and LPS responses, respectively: *red*, increases; *blue*, decreases. *Upper left*, expanded section of **b** and **c**

Joint modules of response to nPM and LPS were identified by WGCNA. The WGCNA for nPM and LPS responses gave 45 gene modules, shown as a cluster dendrogram (Fig. 4a). Of these 45 modules (Fig. 4b), 38 were associated with nPM, 4 modules associated with LPS (Fig. 4c), and 2 modules enriched in both exposures (Fig. 4c, right column). These shared modules were the blue module, composed of increased RNAs (total 2042), and turquoise module, decreased RNAs (total 2612). The relationship of the 45 modules with fold change, as derived by SAM, is seen in Fig. 4d.

In the blue module (increased RNAs), nPM responders had twofold more hub genes (1196/2042) than LPS responders (558/2042). For the shared responses of nPM and LPS, hub genes were 25% (497/2042). The turquoise module (decreased RNAs) with 530 shared hub genes had 2.7-fold more hub genes for nPM (1842/2612) than for LPS (681/2612). The hub genes of the blue and turquoise modules were highly correlated by kME and by fold change (biweight mid-correlation +0.44 for nPM, blue; −0.24 for nPM, turquoise).

#### WGCNA process analysis by Gene Ontology (GO)

**Fig. 5:**
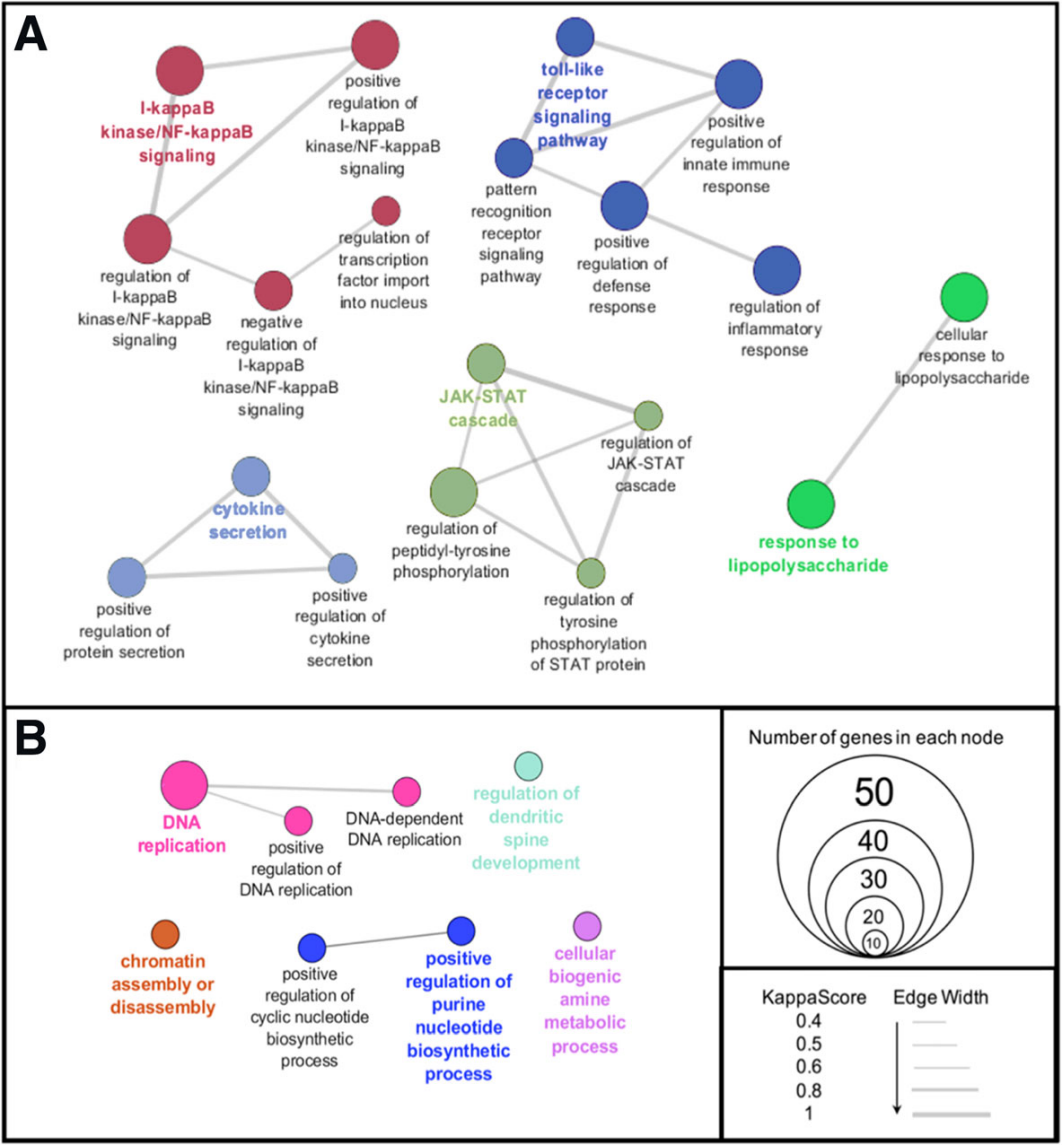
Composition and biological relevance of the two modules enriched by both nPM and LPS. *Blue* (**a**) and *turquoise* (**b**) modules, by GO. Shown above are some select GO processes of interest that are enriched in the two modules. Notably, WGCNA composed the blue module of all increased RNAs, while the turquoise module was decreased RNAs. **a** Many of the pathways observed in the more stringent GO analysis of SAM analysis of nPM are represented in the shared blue module. These include the TLR4 signaling pathway, NF-κB pathway, and inflammatory cytokines. **b** Some similar pathways to decreased RNAs by SAM are observed in the turquoise module, including DNA replication. Nodes with highlighted texted were chosen as the node that best represents the network; node colors distinguish between networks. Nodes with two colors belong to both networks. The circle size represents the number of genes enriched in the node. The width of connecting lines represents the strength of connectivity between nodes

The hub genes of the two shared WGCNA modules, blue and turquoise, were analyzed by GO (Fig. 5a, b, respectively).

The shared hub genes for both nPM and LPS treatment in the blue module (increased RNAs) enriched similar biological processes as the SAM analysis for nPM treatment (Fig. 3a): “I-kappaB kinase/NF-kappaB signaling”, “TLR signaling pathway”, “JAK-STAT cascade”, “cytokine secretion”, and “response to LPS”, among other processes (Fig. 5a). The shared nodes include genes composing the intracellular TLR4 signaling pathway, components of NF-κB, and downstream inflammatory cytokines like TNFα, IL-1α, and IL-1β. All a priori genes of interest (“Background”) were in the blue (increased) module.

The shared hub genes in both nPM and LPS treatment, of turquoise module (decreased RNAs), were enriched for “DNA replication” (also in Fig. 3b), “regulation of dendritic spine development”, “chromatin assembly or disassembly”, “positive regulation of purine nucleotide biosynthetic process”, and “cellular biogenic amine metabolic process” (Fig. 5b).

### Transcription factor DNA binding target (TFT)

Responding RNAs were analyzed for TFTs of their respective genes (Table 1). For nPM, by SAM, there was no enrichment of any TFT. Response to nPM, analyzed by WGCNA, was enriched for BACH1 (regulator of Nrf2 and phase-2 detoxification responses), interferon regulatory factors (IRFs), and other TFTs (Table 1). LPS showed 10 TFTs by SAM, and 8 by WGCNA, with multiple TFTs for interferon regulatory factors (IRFs) by both analyses.

**Table 1:** Transcription factor target (TFT) analysis

Transcription factor	nPM		LPS		Shared	
	SAM	WGCNA	SAM	WGCNA	SAM	WGCNA
AP-1	–	–	1	–	–	–
BACH1	–	1	–	–	–	–
IPF1	–	1	–	–	–	–
IRFs	–	3	6	4	6	4
NFAT	–	1	1	1	–	1
NF-κB	–	–	2	2	5	2
NF1	–	1	–	–	–	–
SRF	–	–	–	–	2	1
STAT1	–	–	–	1	1	–

Numbers represent the total of different TFT binding sequences enriched for each dataset queried. Only the blue and turquoise modules (Fig. 4) were used for WGCNA

*Abbreviations: AP-1* activator protein 1, *BACH1* BT3B and CNC homology 1, *IPF1* insulin promoter factor 1, *IRF* interferon regulatory factor, *NFAT* nuclear factor of activated T cells, *NF–κB* nuclear factor kappa-light-chain-enhancer of activated B cells, *NF1* neurofibromin 1, *SAM* significance analysis of microarrays, *SRF* serum response factor, *STAT1* signal transducers and activator 1

The shared responses showed the largest enrichment of TFTs, with six TFTs for IRFs and five for NF-κB from the RNAs shared by SAM. Analysis by SAM had four and two TFTs for IRF and NF-κB, respectively.

Both IRFs and NF-κB are controlled by intracellular processes following TLR4 activation, suggesting that TLR4 is a mediator of glial responses to nPM and LPS.

### TLR4 siRNA experimental manipulations

**Fig. 6:**
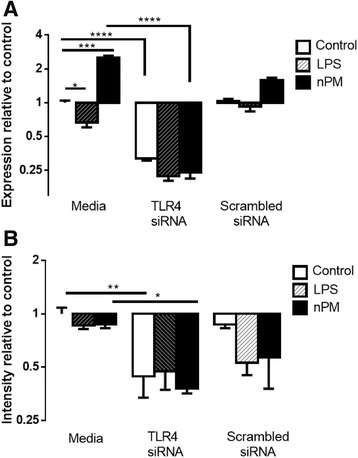
TLR4 response in mixed glia. Mixed glia: TLR4 siRNA treatment lowered TLR4 mRNA and protein, and attenuated nPM induction of TLR4 mRNA. Media groups are non-transfected. **a** TLR4 mRNA, but not protein, was induced by nPM. TLR4 siRNA decreased TLR4 mRNA levels by 90% in nPM (ANOVA, *p <* 0.0001) and 70% in control groups (*p <* 0.0001). LPS treatment reduced TLR4 mRNA (*p <* 0.05). **b** TLR4 protein was not changed by nPM or LPS. TLR4 siRNA reduced protein levels by ca. 60% in all groups (ANOVA, *p <* 0.05)

We verified that TLR4 mRNA was increased 2× by nPM, with no change by LPS (Fig. 6a), whereas TLR4 protein was not altered by nPM or LPS (Fig. 6b). The lack of TLR4 protein induction corroborates findings that LPS, despite activating TLR4, does not increase TLR4 protein by 24 h post treatment [52]. The bioinformatics findings were validated by siRNA manipulation of TLR4.

#### TLR4 knockdown

The siRNA knockdown of TLR4 was confirmed for protein and mRNA. TLR4-siRNA treatment lowered RNA similarly in controls (65%) and nPM (80%) (Fig. 6a). siRNA for TLR4 lowered protein levels by 60% in all groups (Fig. 6b). Scrambled siRNA treatments for TLR4 did not differ from controls (Fig. 6a).

#### Inflammatory proteins

**Fig. 7:**
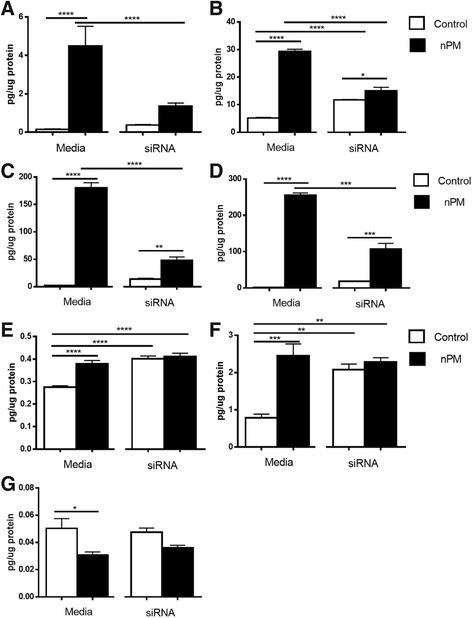
Inflammatory protein panel. Mixed glia: induction of inflammatory cytokines by nPM, with attenuation by TLR4 siRNA. Media groups are non-transfected cultures. **a** TNFα was increased by nPM treatment (*p <* 0.001). siRNA treatment reduced TNFα protein response to nPM (*p <* 0.0001). TNFα protein in siRNA cultures treated with nPM did not differ from controls. **b** IL-1β was increased by nPM (*p <* 0.001). IL-1β induced by nPM was attenuated by TLR4 siRNA (*p <* 0.001). **c** nPM increased IL-6 protein (*p <* 0.0001). TLR4 siRNA reduced nPM IL-6 induction versus non-transfected cultures (*p <* 0.0001). **d** KC protein was increased by nPM in both non-transfected cultures (*p <* 0.0001) and TLR4 siRNA (*p <* 0.001), with attenuated induction in siRNA cultures. **e** IFN-y was increased by nPM (*p <* 0.0001) and by TLR4 siRNA (*p <* 0.0001). **f** IL-5 was increased by nPM (*p <* 0.001), and by siRNA (*p <* 0.01), with no further increase by nPM. **g** IL-4 was decreased by nPM (*p <* 0.05)

nPM treatment increased protein concentration for six of seven investigated proteins (Fig. 7). TLR4 siRNA reduced induction by nPM for TNFα, IL-1β, IL-6, and KC. TLR4 siRNA had no effect on nPM response for IL-5, IL-4, and IFN-y.

The 30-fold increase of TNFα protein by nPM was blocked by siRNA (−80%) (Fig. 7a). TNFα mRNA was also increased eightfold by nPM, and blocked by siRNA (−80%) (see below). IL-1β protein increased by nPM (5.5×) (Fig. 7b). siRNA treatment induced IL-1β (2.3×), with a modest further induction by nPM (1.3×) (Fig. 7b). IL-6 protein increased 80× by nPM, with reduced induction by nPM in siRNA cultures (3.4×) (Fig. 7c). nPM treatment increased KC 142×, with reduced induction by nPM in siRNA cultures (5.8×) (Fig. 7d).

#### TLR4 pathways

**Fig. 8:**
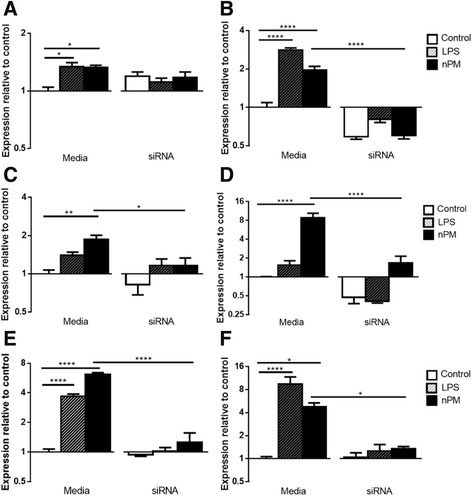
TLR4 pathway activation in mixed glia. Mixed glia: nPM increased mRNAs in the MyD88-dependent TLR4 signaling pathway. Media groups are non-transfected cultures. All values are given as log_2_ fold change. **a** MyD88 was increased by both nPM and LPS (*p <* 0.05), with no change observed in TLR4 siRNA-treated cultures. **b** TRAFD1 mRNA was increased by nPM treatment (*p <* 0.0001) and by LPS treatment (*p <* 0.0001), and rescued by siRNA treatment (*p <* 0.0001). **c** NF-κB mRNA was increased by nPM treatment (*p <* 0.01), and rescued by siRNA treatment (*p <* 0.05). **d** TNFα mRNA was induced by nPM treatment (*p <* 0.0001). siRNA treatment reduced nPM induction of TNFα mRNA (*p <* 0.0001). **e** JAK2 mRNA was increased by nPM and LPS treatment (*p <* 0.0001), and rescued by siRNA treatment (*p <* 0.0001). **f** STAT1 mRNA was increased by nPM and LPS treatment (*p <* 0.01 and *p <* 0.0001, respectively), and rescued by siRNA treatment (*p <* 0.05)

nPM activated the MyD88-dependent TLR4 pathway, but did not activate the MyD88-independent endocytosis pathway. MyD88 was increased by both LPS and nPM (1.3× for both), and blocked by siRNA-TLR4 (Fig. 8a). TRAF6, which is downstream of MyD88-dependent TLR4 activation, did not respond to nPM or LPS (not shown). TAK1, a downstream effector of TRAF6, was also unchanged by nPM or LPS (not shown). TRAFD1, which shows negative feedback on TRAF6, was increased by nPM and LPS (2× and 3×, respectively), blocked by TLR4 siRNA (Fig. 8b).

NF-κB was increased by nPM (2×), with induction blocked by TLR4 siRNA (Fig. 8c). Mentioned above (“Inflammatory proteins”), TNFα was increased 8× by nPM, and blocked by TLR4 siRNA (−80%) (Fig. 8d). TNFR1 mRNA did not respond to nPM or LPS (not shown).

In the JAK/STAT pathway, JAK2 was activated by nPM and LPS (6.1× and 3.7×, respectively), with siRNA blockade (Fig. 8e). STAT1 mRNA was increased by nPM (4.8×) and by LPS (9.1×), and also siRNA blocked (Fig. 8f).

TRIF (Toll/IL-1 receptor domain-containing adaptor protein-inducing IFN-β), of the MyD88-independent endocytosis pathway, was decreased by LPS (0.72×, *p <* .05) and did not respond to nPM (not shown). IFN-β did not respond to nPM or LPS (not shown). This further confirms the absence of endotoxin activity in our nPM, because the “endocytosis pathway” is a known endotoxin-specific TLR4 response.

**Fig. 9:**
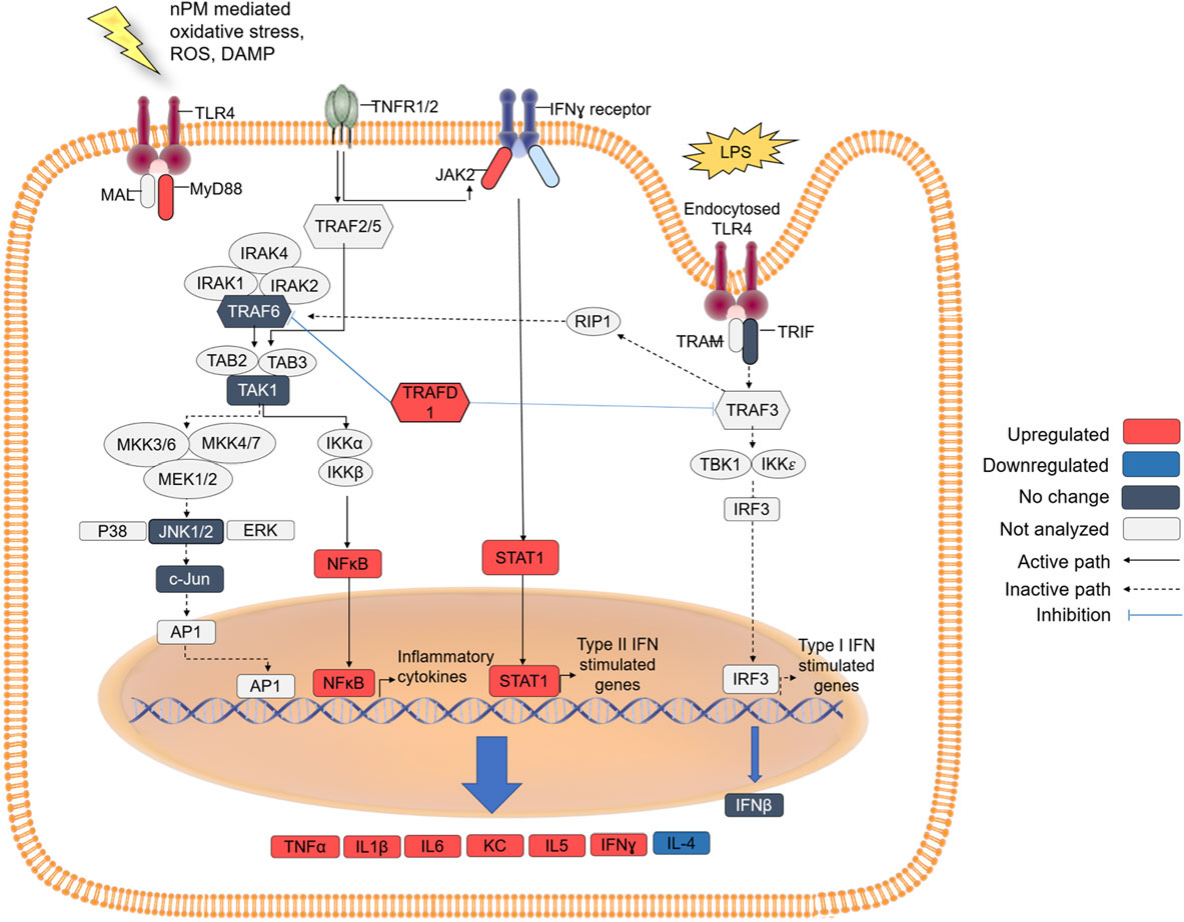
Proposed pathway of TLR4 activation by nPM treatment. *Red*: TLR4-dependent increased mRNA or protein (cytokines). *Dark blue*: proteins or mRNA unresponsive to nPM. *White*: proposed intermediates, not examined. nPM activated MyD88-dependent pathways, increasing NF-κB mRNA and increasing downstream cytokine productions of NF-κB activation. JAK/STAT pathway was also activated by nPM with TLR4 dependence. LPS-mediated TLR4 receptor activation by endocytosis was not altered by nPM treatment

Figure 9 summarizes these responses (“Inflammatory proteins” and “TLR4 pathways”) by pathway.

#### Comparative expression

**Fig. 10:**
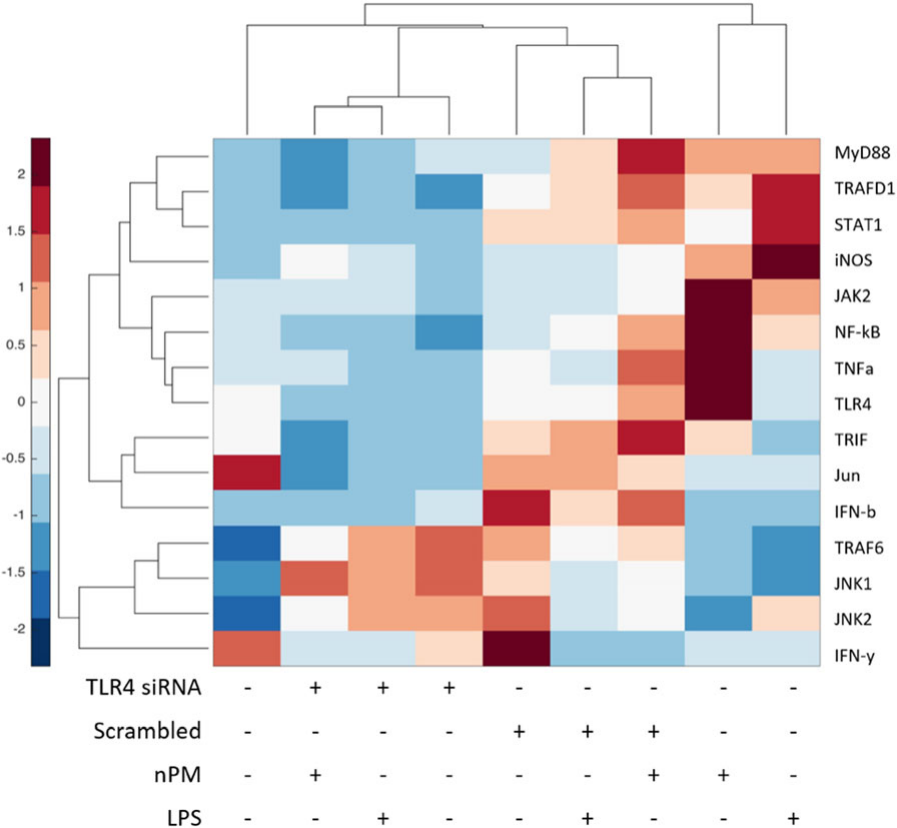
mRNA responses in the TLR4 pathway. Fold change values were standardized, and are shown from *red* (increased) to *blue* (decreased). Genes queried are clustered on the *y*-axis, with genes clustered based on correlation. Treatment groups are clustered on the *x*-axis, with groups showing similar responses clustered closer together

A heatmap of all genes queried by q-PCR in the TLR4 pathway (Fig. 9) shows clusters by genes on the *y*-axis, and treatment groups on the *x*-axis (Fig. 10).

Treatment clustering (*x*-axis): the non-transfected cultures, the control group shows the least similarity to the nPM and LPS groups. The clustering of three TLR4 siRNA groups suggests similar responses. Importantly, on the heat map, the TLR4 siRNA-treated cultures are in the adjacent column to the control group, indicating the siRNA treatment returned RNA to control levels. Gene clustering (*y*-axis): NF-κB, TLR4, and JAK2, cluster with TLR4.

### In vivo nPM exposure

**Fig. 11:**
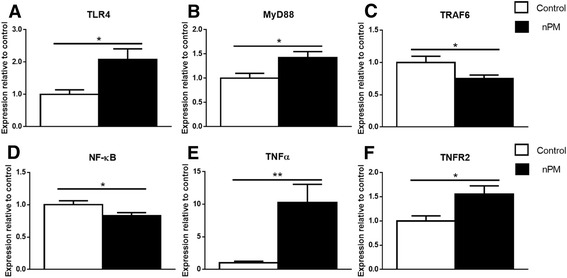
Hippocampal responses to nPM exposure in vivo. Mouse in vivo: Chronic nPM exposure induced components of the TLR4 pathway in hippocampus. mRNA responses by q-PCR are given, shown in relation to pathway (Fig. 7). **a** TLR4 mRNA was induced by nPM (*p <* 0.05). **b** MyD88 mRNA was increased by nPM (*p <* 0.05). **c** TRAF6 mRNA was decreased by nPM (*p <* 0.0001). **d** NF-κB was decreased by nPM (*p <* 0.05). **e** TNFα mRNA was increased by nPM (*p <* 0.01). **f** TNFR2 mRNA was increased by nPM (*p* < 0.05). *n =* 7

To investigate TLR4 involvement in vivo, we examined TLR4-associated responses in the hippocampus of adult mice chronically exposed to nPM for 150 h over 10 weeks. TLR4 and MyD88 RNAs were increased by this nPM exposure (2.1× and 1.4×, respectively) (Fig. 11a, b), while NF-κB and TRAF6 were decreased (0.8× and 0.75×, respectively) (Fig. 11c, d). Increases were observed for TNFα (10×) (Fig. 11e) and TNFR2 (1.6×) (Fig. 11f). There was no response of TNFR1, c-Fos, or c-Jun (not shown).

## Discussion

This is the first comprehensive expression analysis of rodent in vitro glial responses to TRAP particulate matter, which shows broad activation of immune and stress pathways in response to nPM, with 2000 transcripts responding. Guided by the expression analysis, we then demonstrated TLR4 knockdown attenuated a subset of inflammatory response to nPM. Finally, we showed that TLR4 and associated TLR4 pathway RNAs were altered in the hippocampus following in vivo nPM exposure.

This is also the first direct comparison of LPS and air pollution PM for gene expression responses. WGCNA in conjunction with SAM was used to show the extensive overlap of inflammatory responses between nPM and LPS. About 25% of the RNAs responding to nPM were shared with LPS responses. The overlap was enriched for inflammatory responses like TLR4, NF-κB, TNFα, and multiple interleukins. Shared responses to nPM and LPS were also enriched in transcription factor targets (TFTs) for NF-κB and IRFs. These findings are consistent with the exacerbation of lung LPS inflammatory responses when combined with PM exposure [53, 54].

Importantly, the shared LPS and nPM responses were not due to endotoxin presence in these nPM samples. The negligible endotoxin activity of the nPM is consistent with findings that the endotoxin contained within PM is mostly bound in large diameter particles (coarse PM) [33], which are excluded from collection of nPM. Physiologically, the coarse PM are mainly trapped in upper airways.

The nPM and LPS treatments differed 10-fold in the scale of responding RNA modules by WGCNA: nPM treatment enriched 85% (38/45) modules, while LPS enriched only 9% (4/45). This 10-fold greater genomic response to nPM than LPS may be attributed to the greater chemical heterogeneity of the nPM, which includes transition metals, nitrate, sulfate, and water-soluble organic compounds [18].

Glial activation by air pollution exposure is characterized by cell-specific markers like GFAP (astrocytes) and Iba1 (microglia) [18, 23, 55], and by inflammatory cytokines like TNFα and IL-1α [17, 18, 20, 21]. Our results with mixed glia corroborate the cytokine increases, and extend the responses to new signaling pathways including TLR4, NF-κB, and JAK/STAT, as shown by WCGNA (blue module). The enrichment of transcription factors for NF-κB and IRFs by nPM treatment suggests the important role of TLR4 activation in glial responses to nPM.

The TLR4-dependent glial responses to nPM were shown by TLR4 knockdown in vitro, which attenuated the nPM induction of TNFα and other cytokines. This is best visualized by the heatmap (Fig. 10). Non-transfected control and nPM or LPS-treated cultures were on opposite extremes of the heat map, indicating the least similarity of any conditions. Conversely, TLR4 siRNA cultures treated with nPM or LPS were adjacent to control cultures, indicating highest similarity of any treatment groups. Though both nPM and LPS responses are TLR4 dependent, they function through divergent intracellular TLR4 signaling pathways. LPS activates TLR4 through both the TLR4 receptor endocytosis response to endotoxin, and the MyD88-dependent NF-κB inflammatory pathway. The nPM activated the NF-κB inflammatory response through MyD88-dependent signaling, but not the endocytosis pathway. NF-κB activation leads to increased TNFα, IL-1β, and IL-6 (seen in "Inflammatory Proteins") [56]. In vivo exposure of mice to nPM induced hippocampal RNA changes for TLR4 components of MyD88-dependent NF-κB activation, and the TNFα pathway.

The activation of TLR4 with TNFα induction by nPM has implications for the neurodegenerative and cognitive impact of air pollution exposure (see “Background”). We hypothesize that TLR4 activation in glia is a mediator of neurite atrophy induced in young mice by air pollution PM, assessed as the area of silver-stained neuronal processes [8]. In co-cultures of mixed glia and neonatal neurons, nPM impairs neurite outgrowth, mainly via the TNFα secreted by nPM exposed mixed glia [27]. TLR4-knockout mice showed protection from airway inflammation [57], giving a rationale for considering neuroprotection by drugs that block TLR4. However, blocking TLR4 in humans, as a therapeutic approach must be considered cautiously, because attenuated TLR4 function, seen in human TLR4 SNP variants, increases the susceptibility to infection [58].

The direct translocation of nPM into the brain, via the nose, is consistent with the rapid translocation of radiolabeled carbon and manganese ultrafine PM into the brain from the olfactory neurons in the olfactory epithelium [25, 26]. Moreover, we recently showed rapid microglial activation in the olfactory epithelium following nPM exposure, with subsequent cortical neuroinflammation [23].

Other neurodegenerative conditions also involve TLR4 in glial and neuronal responses [59]. In cerebral ischemia/reperfusion models for stroke injury, HSP70 binds to TLR4, activating MyD88-IRAK-TRAF6 and NF-κB pathways, with downstream induction of TNFα and other cytokines [60, 61], as observed for nPM-LPS responses (Fig. 8). The synergies of cerebral ischemic damage with air pollution, observed clinically and in a rodent model [40], may now be understood as mediated by the nPM-LPS TLR4 modules described here and the TLR4 role in cerebral ischemia [59].

The role of TLR4 may extend to pro-amyloidogenic air pollution associations [8, 62–64]. In transgenic mouse AD models, neurodegenerative changes were modulated with varying TLR4 expression [65, 66]. Although no TLR variant has been validated as a risk factor for AD, TLR4-mediated monocyte responses showed individual variations with more than 1000 QTLs [67]. Notably, most of these QTLs involve genes associated with lysosome pathways, consistent with the endosomal associations of nPM-LPS in glial responses. These QTLs may be examined in future studies for SNPs that modify neurotoxic impact of air pollution. We anticipate a broad sharing of inflammatory gene variants that converge on TLR pathways in the neurodegenerative impact of air pollution that accelerates cognitive aging and increases dementia risk.

## Conclusions

This in vitro evidence showed TLR4-dependent *and* independent glial responses to nPM. Knockdown of TLR4 reduced nPM induction of numerous inflammatory cytokines, and attenuated activation of NF-κB and JAK/STAT pathways. Chronic nPM exposure caused TLR4 activation in the hippocampus and increased TNFα, which we hypothesize is a mediator of neurite atrophy in the hippocampus. These results further resolve the mechanisms by which nPM elicits neuroinflammation, and suggest TLR4’s involvement in cognitive impairments from air pollution exposure.

## Additional files

Additional file 1: Gene kME relationship between modules: The two shared modules, enriched by both treatments, had strong inverse correlations between the gene-module specific kMEs, which is the eigengene-based connectivity for a gene within a module (nPM, R = −0.994; LPS, R = −0.968). Each gene is given a kME for every module, and then placed into the module of best fit. Thus, individual RNAs with increased expression had strong inverse associations with the eigengene for the turquoise module, while RNAs with decreased expression had strong inverse associations with the eigengene for the blue module. As examples, TNFα had a kME of +0.93 in the blue module, but −0.91 in the turquoise module; again, the subunits NF-κB1 and NF-κB2 had positive kMEs of +0.91 and +0.96, respectively, in the blue module, but negative kMEs of −0.86 and −0.93, respectively, in the turquoise module. Together this suggests that the turquoise and the blue modules represent a single network, where the turquoise comprises the decreased RNAs in the network and the blue comprises the increased RNAs. (ZIP 43372 kb)

Additional file 2: Figure S1. A, LPS-increased RNAs: clustered into networks. 16 nodes, composing 6 networks and 3 individual nodes, are depicted. B, LPS-decreased RNAs: two single nodes are depicted. Colors denote different networks. Nodes with two colors belong to both networks. Each network has one highlighted node (colored text, chosen by experimenter) that best represents network function. The circle size represents the number of genes enriched in the node. The width of connecting lines represents the strength of connectivity between nodes, as measured by kappa score. (ZIP 347 kb)

## Abbreviations

AP-1: Activator protein 1
BACH1: BT3B and CNC homology 1
BACH1: BTB domain and CNC homolog 1
c-Jun: Jun proto-oncogene
EU: Endotoxin units
Fos: Fos proto-oncogene
GAPDH: Glyceraldehyde 3-phosphate dehydrogenase
GO: Gene Ontology
IFN-y: Interferon gamma
IFN-β: Interferon beta
IL: Interleukin
IPF1: Insulin promoter factor 1
IRAK: Interleukin 1 receptor-associated kinase 1
IRF: Interferon regulatory factor
JAK2: Janus kinase 2
JNK1/2: c-Jun N-terminal kinase
KC: Chemokine (C-X-C motif) ligand 1
KEGG: Kyoto Encyclopedia of Genes and Genomes
kME: Eigengene-based connectivity
LPS: Lipopolysaccharide
MyD88: Myeloid differentiation primary response 88
NF1: Neurofibromin 1
NFAT: Nuclear factor of activated T cells
NF-κB: Nuclear factor kappa-light-chain-enhancer of activated B cells
nPM: Nanoscale particulate matter
PM: Particulate matter
QTL: Quantitative trait loci
SAM: Significance analysis of microarrays
SRF: Serum response factor
STAT1: Signal transducers and activator 1
TAK1: Transforming growth factor beta-activated kinase 1
TFT: Transcription factor target
TLR4: Toll-like receptor 4
TNFR1: TNFα receptor 1
TNFRSF9: TNF receptor super family 9
TNFα: Tumor necrosis factor alpha
TOM: Topological overlap matrix
TRAF3ip: TNF receptor-associated factor 3 interacting protein
TRAF6: TNF receptor-associated factor 6
TRAFD1: TNF receptor-associated factor domain 1
TRAP: Traffic-related air pollution
TRIF: Toll/IL-1 receptor domain-containing adaptor protein-inducing interferon beta
WGCNA: Weighted gene co-expression network analysis
