# Supplementary material for: Toll-like receptor 4 in glial inflammatory responses to air pollution in vitro and in vivo
Source: J Neuroinflammation. 2017 Apr 14;14:84. doi: 10.1186/s12974-017-0858-x (PMC5391610; doi:10.1186/s12974-017-0858-x)
Supplement: Supplementary file 2 — A, LPS-increased RNAs: clustered into networks. 16 nodes, composing 6 networks and 3 individual nodes, are depicted. B, LPS-decreased RNAs: two single nodes are depicted. Colors denote different networks. Nodes with two colors belong to both networks. Each network has one highlighted node (colored text, chosen by experimenter) that best represents network function. The circle size represents the number of genes enriched in the node. The width of connecting lines represents the strength of connectivity between nodes, as measured by kappa score. (ZIP 347 kb) [file 12974_2017_858_MOESM2_ESM.zip › Supplementary information 2-28-17.docx]

**Title: Toll-like Receptor 4 in Glial Inflammatory Responses to Air Pollution *in Vitro* and *in Vivo*.**

Running Title: Glial inflammation by air pollution TLR4 dependent

**Nicholas C. Woodward^1^, Morgan C. Levine^2^, Amin Haghani^1^, Farimah Shirmohammadi^3^, Arian Saffari^3^, Constantinos Sioutas^3^, Todd E. Morgan^1^, Caleb E. Finch^1,4*^.**

1, Leonard Davis School of Gerontology, University of Southern California, Los Angeles, CA.

2, Dept. of Human Genetics, David Geffen School of Medicine, University of California Los Angeles, Los Angeles, CA.

3, Viterbi School of Engineering, University of Southern California, Los Angeles, CA.

4, Dornsife College, University of Southern California, Los Angeles, CA.

*Corresponding author. Email [cefinch@usc.edu](mailto:cefinch@usc.edu).

**Woodward NC** [**nwoodwar@usc.edu**](mailto:nwoodwar@usc.edu) **; Levine, MC** [**morgan.elyse.levine@gmail.com**](mailto:morgan.elyse.levine@gmail.com)**;**

**Haghani A** [**ahaghani@usc.edu**](mailto:ahaghani@usc.edu) **; Shirmohammadi F** [**shirmoha@usc.edu**](mailto:shirmoha@usc.edu)**;**

**Saffari A** [**asaffari@usc.edu**](mailto:asaffari@usc.edu)**; Sioutas C sioutas@usc.edu;**

**Morgan TE** [**temorgan@usc.edu**](mailto:temorgan@usc.edu) **;Finch CE cefinch@usc.edu**

**Supplementary information:**

Gene kME Relationship Between Modules: The two shared modules, enriched by both treatments, had strong inverse correlations between the gene-module specific kMEs, which is the eigengene-based connectivity for a gene within a module (nPM, R= -0.994; LPS, R= -0.968). Each gene is given a kME for every module, and then placed into the module of best fit. Thus, individual RNA’s with increased expression had strong inverse associations with the eigengene for the turquoise module, while RNAs with decreased expression had strong inverse associations with the eigengene for the blue module. As examples, TNFα had a kME of +0.93 in the blue module, but -0.91 in the turquoise module; again, the subunits NF-κB1 and NF-κB2 had positive kME’s of +0.91 and +0.96, respectively, in the blue module, but negative kME’s of -0.86 and -0.93, respectively, in the turquoise module. Together this suggests that the turquoise and the blue modules represent a single network, where the turquoise comprises the decreased RNAs in the network and the blue comprises the increased RNA’s.

**Supplementary Figure 1**:

A, LPS-increased RNA’s: clustered into networks. 16 nodes, composing 6 networks and 3 individual nodes, are depicted.

B, LPS-decreased RNA’s: two single nodes are depicted.

Colors denote different networks. Nodes with two colors belong to both networks. Each network has one highlighted node (colored text, chosen by experimenter) that best represents network function. The circle size represents the number of genes enriched in the node. The width of connecting lines represents the strength of connectivity between nodes, as measured by kappa score.

**Supplementary Table 1. Verification of microarray changes by qPCR**

| Gene name | Microarray fold change nPM | q-PCR  nPM | Microarray fold change LPS | q-PCR LPS |
| --- | --- | --- | --- | --- |
| Increased | | | | |
| JAK2 | 2.7 | 5.1 | 4.2 | 3.3 |
| STAT1 | 4.5 | 3.5 | 2.0 | 2.5 |
| TNFa | 2.2 | 19 | 3.3 | 9.9 |
| TNFRsf9 | 5.9 | 26 | 2.3 | 22 |
| TRAFD1 | 2.7 | 3.1 | 1.6 | 4.7 |
| Decreased | | | | |
| BRCA | 0.8 | 0.4 | 1.0 | 0.6 |
| Unchanged | | | | |
| Fos | 0.6 | 0.9 | 0.2 | 1.1 |
| TRAF3ip | 0.8 | 1.1 | 1.5 | 1.1 |
| TRAF6 | 1.2 | 1.5 | N/A | 1.4 |

Genes queried by q-PCR,to verify microarray analysis showed consistent trends for all genes. Significant increase in JAK2, STAT1, TNFa, TNFRsf9, and TRAFD1. BRCA was decreased, while Fos and TRAF3IP and TRAF6 were unchanged. N= 4 per microarray group; N= 14 per q-PCR group. A random subset was chosen for microarray analysis. Abbreviations: BRCA, breast cancer, Fos, FBJ murine osteosarcoma viral oncogene homolog; JAK2, janus kinase 2; STAT1, signal transducer and activator of transcription 1; TNFa; TNFRSF9, TNF receptor super family 9; TRAFD1, TNF receptor associated factor zinc finger domain containing 1; TRAF3IP, TNF receptor associated factor 3 interacting protein; TRAF6, TNF receptor associated factor 6.
