# Supplementary figures and images for: Toll-like receptor 4 in glial inflammatory responses to air pollution in vitro and in vivo
Source: J Neuroinflammation. 2017 Apr 14;14:84. doi: 10.1186/s12974-017-0858-x (PMC5391610; doi:10.1186/s12974-017-0858-x)

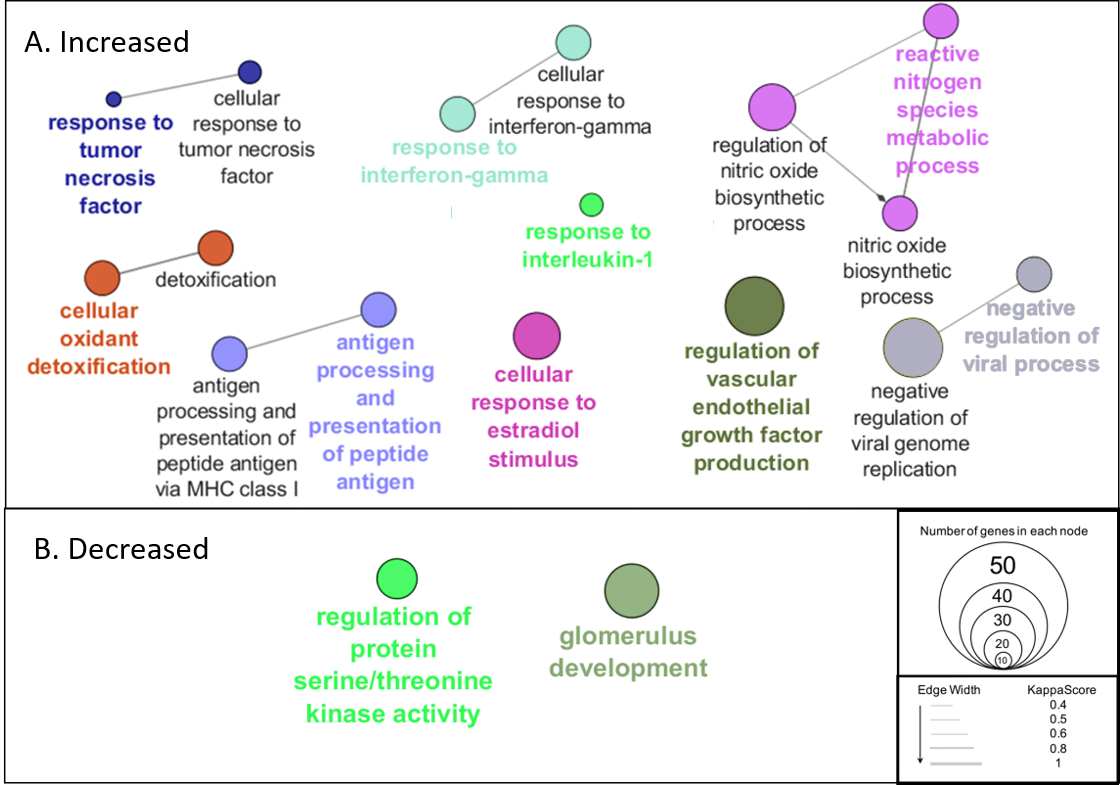

Supplement: Supplementary file 2 — A, LPS-increased RNAs: clustered into networks. 16 nodes, composing 6 networks and 3 individual nodes, are depicted. B, LPS-decreased RNAs: two single nodes are depicted. Colors denote different networks. Nodes with two colors belong to both networks. Each network has one highlighted node (colored text, chosen by experimenter) that best represents network function. The circle size represents the number of genes enriched in the node. The width of connecting lines represents the strength of connectivity between nodes, as measured by kappa score. (ZIP 347 kb) [file 12974_2017_858_MOESM2_ESM.zip › Supplementary Figure 1 2-28-17.png]
